# Isomer-Resolved Imaging of Prostate Cancer Tissues Reveals Specific Lipid Unsaturation Profiles Associated With Lymphocytes and Abnormal Prostate Epithelia

**DOI:** 10.3389/fendo.2021.689600

**Published:** 2021-08-05

**Authors:** Reuben S. E. Young, Britt S. R. Claes, Andrew P. Bowman, Elizabeth D. Williams, Benjamin Shepherd, Aurel Perren, Berwyck L. J. Poad, Shane R. Ellis, Ron M. A. Heeren, Martin C. Sadowski, Stephen J. Blanksby

**Affiliations:** ^1^School of Chemistry and Physics, Queensland University of Technology, Brisbane, QLD, Australia; ^2^M4I, The Maastricht MultiModal Molecular Imaging Institute, Division of Imaging Mass Spectrometry, Maastricht University, Maastricht, Netherlands; ^3^Australian Prostate Cancer Research Centre - Queensland, Faculty of Health, Queensland University of Technology, Princess Alexandra Hospital, Translational Research Institute, Brisbane, QLD, Australia; ^4^Department of Pathology, Princess Alexandra Hospital, Brisbane, QLD, Australia; ^5^Institute of Pathology, University of Bern, Bern, Switzerland; ^6^Central Analytical Research Facility, Queensland University of Technology, Brisbane, QLD, Australia; ^7^Molecular Horizons and School of Chemistry and Molecular Bioscience, University of Wollongong, Wollongong, NSW, Australia; ^8^Illawarra Health and Medical Research Institute (IHMRI), University of Wollongong, Wollongong, NSW, Australia

**Keywords:** imaging, mass spectrometry imaging, lipid, pathology, lipid metabolism

## Abstract

Prostate cancer is the fourth most common cancer worldwide with definitive diagnosis reliant on biopsy and human-graded histopathology. As with other pathologies, grading based on classical haematoxylin and eosin (H&E) staining of formalin fixed paraffin-embedded material can be prone to variation between pathologists, prompting investigation of biomolecular markers. Comprising around 50% of cellular mass, and with known metabolic variations in cancer, lipids provide a promising target for molecular pathology. Here we apply isomer-resolved lipidomics in combination with imaging mass spectrometry to interrogate tissue sections from radical prostatectomy specimens. Guided by the histopathological assessment of adjacent tissue sections, regions of interest are investigated for molecular signatures associated with lipid metabolism, especially desaturation and elongation pathways. Monitoring one of the most abundant cellular membrane lipids within these tissues, phosphatidylcholine (PC) 34:1, high positive correlation was observed between the n-9 isomer (site of unsaturation 9-carbons from the methyl terminus) and epithelial cells from potential pre-malignant lesions, while the n-7 isomer abundance was observed to correlate with immune cell infiltration and inflammation. The correlation of lipid isomer signatures with human disease states in tissue suggests a future role for isomer-resolved mass spectrometry imaging in assisting pathologists with prostate cancer diagnoses and patient stratification.

## Highlights

- Tissue imaging using isomer-resolved mass spectrometry reveals significant variation in lipid unsaturation across prostate cancer tissues
- Increased fractions of PC 34:1*n*-9 are associated with abnormal prostate epithelial cells
- Increased fractions of PC 34:1*n*-7 are associated with immune cell infiltration and inflammation
- Changes in lipid isomer ratios are suggested as sensitive markers for metabolic change within prostate cancer and altered tissue cellular composition
- Calibration using external standards provides a mole ratio of lipid isomers

## Introduction

Prostate cancer is the second most common cancer within men and the fourth most common cancer overall (1). Although prostate specific antigen levels and magnetic resonance imaging can provide an indication of prostate cancer onset and unusual growth, prostate biopsy and Gleason grading *via* histopathology remains the sole means of establishing a definitive cancer diagnosis (2, 3). Given the existence of several benign mimickers of malignant prostate cancer, the limited number of biopsy needle cores available for diagnostic interpretation, the biopsy examination being heavily reliant on human perception, and the importance of pathologist expertise specifically in reviewing prostate tissue, misdiagnoses are relatively frequent and thus subsequent collections are often required for confirmation (4, 5). Indeed, a histopathological study of prostate specimens from 1359 men (5) showed that a minimum of 30 false-positive diagnoses were recorded (≥2.2%) when assessed with strict inclusion criteria, with misinterpretation of benign prostatic features (*e.g.*, adenosis, partial atrophy, prostatic intraepithelial neoplasia) or normal anatomy (seminal vesicles) being the cause of misdiagnosis. Recently, there has been an interest in developing computerised deep learning protocols to assist pathologists with the workload brought on by increasing prostate cancer prevalence (6). These protocols however, still rely on analysis of stained tissue sections to identify cancer and grade tumorous tissues. Therefore, there exists a need for a more quantifiable molecular pathology approach to assist histopathologists in distinguishing benign and malignant features, and thus, improving diagnostic accuracy and turnaround time.

An ideal target for molecular pathology analyses needs to be morphologically informative, specific to clinically significant malignant cells, and easily detectable. Within eukaryotes, lipids comprise approximately 50% of cellular mass and serve various functional roles in energy storage, membrane structure and signalling (7). Because these processes are central to cell division, sustained proliferation induced by cancer (amongst other hallmarks) (8) will inevitably affect lipid populations. One of the most abundant lipid classes that influence both membrane structure and signaling are the glycerophospholipids (GP). GPs are amphoteric molecules that consist of a hydrophilic head group linked, *via* a glycerol backbone, to one or two fatty acids. Because of their distinct biosynthetic origins, functional diversity and abundance, GPs are an ideal candidate for molecular pathology strategies.

One approach to molecular pathology that is currently receiving increased interest from the medical community is matrix-assisted laser desorption/ionization mass spectrometry (MALDI-MS), which uses a focused laser to sample distinct regions of tissue (or other substrates) (9). When a tissue section is rastered under the laser, images can be generated that map the distribution and abundance of ionized molecules in a method known as mass spectrometry imaging (MSI). Using MALDI-MSI to monitor the abundance of ischemia-associated GPs and their degradation products, such as cardiolipin to lysocardiolipin, van Smaalen et al. were able to rapidly assess the extent of ischemic injury in renal tissue to evaluate organ-transplant viability (10). In contrast, histopathology was unable to statistically differentiate ischemic injury from healthy tissue. There are however limitations to imaging GPs *via* MALDI-MSI. One such limitation arises from the inherently different acid-base properties among the GPs that leads to preferential ionization of some lipids in positive ion mode, while others are more readily detected in negative ion mode. This can result in an incomplete representation of GP speciation and distribution across a tissue unless the experiment is run using dual-polarity sampling, which in turn increases the duration of acquisition (11). Similarly, ionisation suppression can also lead to class-based quantitative biases when using MALDI-MS, which can be partially resolved by using a post-ionisation method such as MALDI2 (12). Although this can be reasonably well achieve at a molecular-level (13), another significant challenge is the reliable identification of isomeric lipids in MALDI-MS spectra, acquired directly from tissue. With many isobaric (same nominal mass) and isomeric (same elemental composition) lipids known to be present within the tissues and cells, higher mass resolution (14) and additional tandem mass spectrometric techniques are required for accurate species identification (15).

While there are a number of different GP headgroups, the fatty acids (FA) linked to the glycerol backbone are even more diverse in molecular structure and include numerous isomeric forms. Consisting of a carboxylate group and a hydrocarbon chain, mammalian fatty acids usually consist of between 14 and 24 carbons, with the vast majority containing either 16 or 18 carbons. Although initially biosynthesized as saturated chains (*i.e.*, no double bonds), fatty acids (as fatty acyl-CoA) can act as substrates for desaturase enzymes, resulting in a point of unsaturation (*i.e.*, a carbon-carbon double bond) at a specific location (Δ-position) relative to the carboxylate moiety. Because of the regiospecificity of desaturase enzymes, the position of the double bond (DB) relative to the methyl terminus (*i.e.*, the *n-* number) is dependent on the length of the fatty acid substrate undergoing desaturation. For example, stearoyl CoA desaturase-1 (SCD-1) will install a double bond at the Δ9 position, however depending on if the substrate is either palmitic acid (FA 16:0, *i.e.*, 16 carbons, zero double bonds) or stearic acid (FA 18:0), SCD-1 activity will result in the formation of FAs 16:1*n*-7 or 18:1*n*-9, respectively. Monounsaturated fatty acid products arising from desaturation can then either undergo a two-carbon extension catalyzed by one of a range of elongase isoforms (ELOVL1-7) or a two-carbon loss through peroxisomal or mitochondrial *β*-oxidation yielding a range of double bond isomers (*e.g.*, isomers FAs 18:1*n*-7 and 18:1*n*-9 or isomers FAs 16:1*n*-7 and 16:1*n*-9). Identification and quantification of these fatty acid isomers thus presents a snapshot of cellular metabolism by providing information about lipid-enzyme interactions occurring in that location (16). Furthermore, identification of fatty acid isomers can infer molecular functions and signalling. For example, oleic acid (FA 18:1*n*-9) works alongside angiotensin II to induce synergistic mitogenic responses in vascular smooth muscle cells through protein kinase activation (17), while its *β*-oxidation product FA 16:1*n*-9 has been shown to exhibit an anti-inflammatory effect equivalent to that of docosahexaenoic acid (FA 22:6*n-*3) (18, 19). Likewise, FA 16:1*n*-7 (a lipokine) was found to regulate SCD-1 expression (20), while uptake of dairy-derived FA trans-DB isomers 16:1*n*-7(*trans*) and 18:1*n*-7(*trans*) have been associated with lower diabetes rates (21) and increase concentrations of endocannabinoids (affecting metabolic regulation, smooth muscle contraction and inflammation) and noncannabinoids (affecting PPARα activity, lipolysis and inflammation) (22). These examples highlight the importance of discriminating between fatty acid double bond isomers when characterizing and mapping GPs in a biological context.

Discriminating between lipid DB isomers presents a significant challenge for mass spectrometry due to their identical empirical formulae (and thus identical mass-to-charge ratios, *m/z*) and indistinguishable fragmentation patterns upon collision-induced dissociation. To overcome this challenge, bespoke mass spectrometric methods have been developed (23–27), including a technique known as ozone induced dissociation (OzID) (28). This ion-activation approach utilizes the gas-phase ion-molecule reaction between ozone and ionized lipids within the mass spectrometer. The chemical reaction facilitates chemo-selective cleavage of carbon-carbon double bonds within the acyl chain to yield product ions that are characteristic of the double bond(s) location(s). Pairing OzID with MALDI-MSI then allows for lipid double bond isomer distributions to be mapped across a tissue, in turn revealing a layer of information previously unavailable to conventional lipidomic analysis. Mass spectrometry imaging using a MALDI-OzID modality was first demonstrated by Paine et al., who showed distinctive distributions of the phosphatidylcholine isomers PC 34:1*n*-7 and PC 34:1*n*-9 across murine brain tissue, revealing features that were either invisible or poorly visualized by immunochemical staining (29). Given our recent observations of distribution differences between lipid isomers across human prostate tissues (16), here we investigate the distribution of PC 34:1 double bond isomers across human prostate tumor tissue and explore correlations with the morphological tissue features determined by histopathology grading. Using a set of high purity lipid isomers (>97% regiopurity), we develop calibrations to correct for detection bias between isomers and provide relative abundance values for the lipid isomers in regions of interest. These mole fractions then allow for lipid isomer changes to be observed between tissue samples without the need to calibrate to an internal standard. Applying this calibration to MALDI-MSI-OzID of prostate tissue, here we show isomer abundance differences occurring within specific tissue types and how these changes may assist and inform clinical pathologists.

## Methods

### Materials

IsoPure^®^ PC standards: PC 16:0/18:1*n*-7 ((cat.# 792455), PC 16:0/18:1*n*-9 (cat.# 792453), PC 18:1*n*-7/16:0 (cat.# 792456) & PC 18:1*n*-9/16:0 (cat.# 792458) were obtained from Avanti Polar Lipids, Alabaster, AL, USA. Methanol (LC-MS grade), acetonitrile (ACN; Optima^®^), water (Optima^®^), isopropanol (IPA; Optima^®^ LC-MS grade) for lipid LC-MS were obtained from Fisher Scientific, Scorseby, VIC, Australia. Ammonium acetate for lipid LC-MS was obtained from Sigma-Aldrich, North Ryde, NSW, Australia. For MALDI-MS related sample prep and analysis, sodium acetate (≥99%), 2,5-dihydroxyacetophenone (DHA; ≥99.5%, Ultra pure), methanol (LC-MS grade) and chloroform (HPLC grade) were obtained from Sigma Aldrich, Zwijndrecht, The Netherlands.

### Biological Sample Preparation

#### Tissue Ethics, Sectioning and Mounting

The prostate tissues used throughout were obtained from radical prostatectomy with the ethical approval of the St. Vincent’s Hospital Human Ethics Committee and in accordance with Australian National Health and Medical Research Council Guidelines. Tissues samples were collected by a pathologist, snap-frozen using liquid nitrogen, and stored at -80°C prior to sectioning. Tissue biopsies were sectioned at 5 μm for H&E staining and 10 μm thickness for lipid analyses using a CM 1950 Cryostat (Leica Biosystems, Nussloch, Germany), and using a blade that was free from optimal cutting temperature (OCT) compound. Tissue sections were mounted on standard glass slides (SuperFrost Plus, Menzel-Gläser, Braunschweig, Germany) and fixed using 10% neutral buffered formalin for 30 s before MALDI-MSI-OzID protocol (see method section *Mass Spectrometry Imaging of Lipid Isomers*) and the procedure used for H&E staining (see method section *Haematoxylin and Eosin Staining*).

#### Haematoxylin and Eosin Staining

After sectioning, mounting, and fixing of prostate tissues, H&E staining was achieved using an autostainer (Tissue-Tek Prisma, Sakura Finetek, Torrance, CA, USA). Tissues were first washed with water for 2 mins before being exposed to haematoxylin (Harris Haematoxylin (PAH), Australian Biostain P/L, Traralgon, Australia) for 5 mins. Nuclear differentiation was achieved with water rinsing for 4 mins and exposure to ethanol (Chem Supply Gilman, Australia) acidified with 1% HCl for 10 s. Further rinsing with water (5 min) occurred prior to 2 mins of eosin staining (0.25% Eosin Y; certified C.C. # 45380, ProSciTech, Kirwan, Australia) before a further 40 s of water rinsing. One 80% ethanol rinse followed by two 100% ethanol rinses then took place for 45, 30 and 45 s, respectively. Triplicate xylene (Point Of Care Diagnostics, North Rock, Australia) washes were then conducted for 1 min each. After coverslips (Tissue-Tek Glas, Sakura Finetek, Torrance, CA, USA) were placed, a Pannoramic Digital slide scanner (3DHistech, Hungary) was used to obtain images for histopathology analyses.

#### Histopathology Analyses

H&E stained sections were reviewed for the presence of prostate cancer by an anatomical pathologist (B.S.), Royal College of Pathologists of Australia Faculty of Science Fellow (E.W.), and the University of Bern’s Director of Pathology (A.P.). Where cancer was present it was graded using the Gleason Grading system (30).

### Analytical Methods

#### Mass Spectrometry Imaging of Lipid Isomers

After sectioning, slides for MALDI-MSI were placed into a sealed slide holder, purged with nitrogen gas and stored on dry ice for inter-laboratory shipping. Samples were stored at -80°C upon arrival. Prior to analysis, tissue sections were first thinly coated with 12 passes (45 mm spray height, 30°C, 10 psi, 2 mm track spacing) of 100 mM sodium acetate dissolved in 2:1 methanol/chloroform using an HTX TM-Sprayer (HTXImaging, Chapel Hill, NC, USA). 2,5-dihydroxyacetophenone (DHA) was then sublimated (40 mg, 160°C, 4 mins) to the sample slides using a sublimator (HTXImaging, Chapel Hill, NC, USA). Slides were then loaded into a prototype μMALDI source (31) for sample desorption and ionisation (Nd : YAG laser operating at 1.5 kHz, producing pulses at 355 nm; Waters, Wilmslow, UK). Using a 1.62 A laser diode current, tissues were sampled with a 50 μm^2^ pixel size at a velocity of 2.0 mm/s. Mass analysis and ozonolysis was undertaken using a SYNAPT G2-S*i* HDMS mass spectrometer (Waters, Wilmslow, UK) modified with a closed loop ozone generation system to deliver ozone (~18% w/w in oxygen) to the travelling-wave ion-mobility cell, as described previously (32). Using oxygen as a feed gas (99.999% purity, Linde Gas Benelux BV, The Netherlands) ozone was generated by a high concentration ozone generator (TG-40; Ozone Solutions, Hull, IA, USA), with the concentration being measured online using a UV-absorption based ozone monitor (106-H, 2B Technologies, Boulder, CO, USA). Precursor ions of *m/z* 782 (including both [PC 34:1 + Na]^+^ and [PC 36:4 + H]^+^ ions) were mass-selected using an isolation width of *ca.* 1 Da. Ion-mobility mode was selected on the instrument to provide ~15 ms of reaction time for ionized lipids with ozone during transit through the ion-mobility region. The resulting monoisotopic ions (precursors and products) were mass analyzed by the reflectron time-of-flight, using the “sensitivity mode” instrument setting (a mass-resolving power of ~15,000).

#### Detection Response Calibration for Lipid Isomers

Two sets of mixtures of synthetic PC 34:1 isomer standards were prepared (IsoPure^®^, Avanti Polar Lipids, Alabaster, AL, USA). Each set included double bond isomers with the monounsaturated 18:1 acyl chain at the canonical (*sn-*2) position (*i.e.*, PC 16:0/18:1*n*-7 and PC 16:0/18:1*n*-9) and non-canonical (*sn-*1) position (i.e., PC 18:*1n*-7/16:0: PC 18:1*n*-9/16:0). Five ratio-metric isomer mixtures for each set with the total lipid concentration of each sample was maintained at 5 µM (in methanol). The concentration ratio of *n*-7: *n*-9 isomers was varied to yield relative proportions of 100:0, 75:25, 50:50, 25:75 and 0:100. These reference standards were analyzed by liquid-chromatography mass spectrometry (LC-MS) with mass spectrometry conditions closely mimicking those employed in MALDI-MS (see above). Measurements were undertaken using a Waters ACQUITY UPLC system coupled with an electrospray ionisation enabled SYNAPT G2-S*i* HDMS mass spectrometer (Waters, Wilmslow UK) modified with an identical OzID system as described above. 1 µL injections of lipid standard mixtures were chromatographically separated using a AQUITY UPLC CSH C18 2.1x100 mm 1.7 µm column (Waters, Wilmslow UK), a column oven temperature of 60°C and a solvent flow rate of 0.4 mL/min. The solvent system used consisted of solvent A) 30% acetonitrile (v/v/v), 20% isopropanol (v/v/v), 50% aqueous ammonium acetate (10 mM) (v/v/v) and solvent B) 9% acetonitrile (v/v/v), 90% isopropanol (v/v/v), 1% aqueous ammonium acetate (10 mM) (v/v/v). A stepped linear gradient was used for analyte elution, the details of which can be seen in **Figure 4**. Column eluents were then ionized *via* electrospray ionisation (spray voltage of 2.5 kV) before entering the vacuum of the mass spectrometer. The quadrupole mass filter was set to transmit all ions (referred to as MS^e^ in the instrument control software), and ions were exposed to ozone as they passed through the ion-mobility cell (approximately 15 ms of ozone exposure). Precursor and characteristic OzID product ions were then mass analyzed using the reflectron time-of-flight with a mass-resolving power of 15,000.

### Quantification and Statistical Analysis

For calibration of the instrument response for lipid isomers, the LC-MS data (*cf.* **Figure 4**) were integrated over the two PC 34:1 chromatographic peaks, and the product ion abundance relating to the *n*-7 and *n*-9 isomers were extracted for each isomeric mixture sample. Intensities (*I*) of the OzID peaks corresponding to one isomer were then divided by the total OzID product peak intensities for both isomers to generate the signal response as a fraction of the OzID product ion signal (*e.g.*, fraction of the PC 34:1(*n-*9) isomer = *I*_(_ *_n_*_-9)_/[*I*_(_ *_n_*_-9)_ + *I*_(_ *_n_*_-7)_]). These response factor values were then plotted such that the y-axis represented the response factor and the x-axis represented the known concentration percentage for each of the five ratiometric dilutions. A 2^nd^-order polynomial trendline was then fitted to the points to obtain an equation to the line. The standard error at the y-intercept was approximately 3 times (2.83) the value of the y-intercept and thus y intercepts were not forced through zero, making the calibration model only accurate for fractions greater than the standard error (S_e_) of 0.022. Regression analysis revealed an R^2^ value of 0.9964 and a S_e_ of 0.022 presenting an accurate model for correcting instrument responses to fractional molar contributions. At a pixel size of 50 µm x 50 µm, two regions of interest (roughly 300 pixels or 300 averaged spectra) from the same section of prostate tissue were examined from the MALDI-MSI-OzID data. Isomer response factors were extracted and subsequently corrected using the calibration curve. Analysis revealed that MS response was not statistically different from corrected molar contributions. In order to attain statistical significance and associated error between the uncorrected ROIs, the spectra of nine 6x6 pixel regions were sampled to align with the cell types contained within each. In addition to these regions, other similar features within the same tissue (as well as features from a separate patient tissue) were sampled for statistical analysis. A two-tailed paired *t*-test was applied to both raw instrument response data and corrected mole fraction data to discern *t*-values. Subsequently, these were converted to p-values (*p ≤ 0.05, **p ≤ 0.01, ***p ≤ 0.001 and ****p ≤ 0.0001) using conventionally accepted critical values of *t* and relevant degrees of freedom. For visual representation of the ROI sampling regions, see **Figure S2**. Division of tissue ROIs into smaller cell ROIs (*cf.* **Figure S2**) also allowed for the standard error to the mean (SEM) to be calculated and is displayed in the figure caption of **Figure 3C**. Error analysis of post-corrected data in **Figure 3D** is produced from the calibration curve S_e_ value.

## Results

### Classification of Prostate Tissues Using H&E Staining and MALDI-MSI-OzID

Two adjacent frozen sections of prostate tissue obtained from radical prostatectomy specimens were mounted on glass slides. The first section (5 µm) underwent H&E staining, followed by the identification of tissue components and states by microscopy. The adjacent section (10 µm) was prepared for MALDI-MSI-OzID, where PC 34:1, one of the most abundant GPs within human prostate tissue, was targeted for isomer resolution. [PC 34:1 + Na]^+^ ions desorbed directly from the tissue by MALDI were mass-selected using an isolation window of 1 Th. It should be noted that although the monoisotopic target ions were selected under these conditions, the nominal *m/z* 782 includes numerous isobaric and isomeric lipids. According to LIPID MAPS Structure Database (LMSD) (33), *m/z* 782.5670 ± 0.0500 (62 Δppm) could comprise up to 30 unique protonated and sodiated lipid species, including the [M + H]^+^ ion of another abundant tissue glycerophospholipid, PC 36:4.

**Figure 1:**
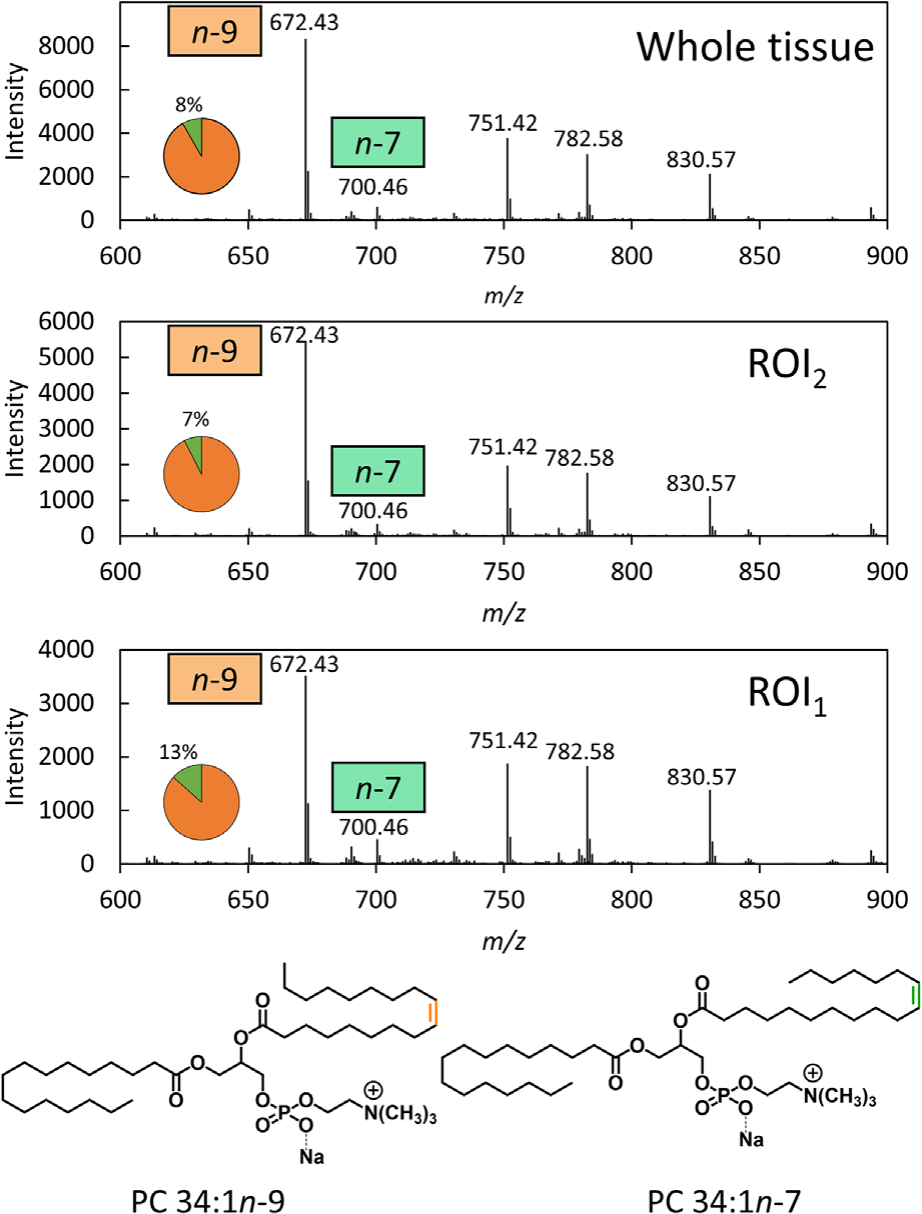
OzID mass spectra averaged across a whole prostate tissue sample (32500 pixels) and 2 individual regions across the tissue (300 pixels each). OzID mass spectra obtained from averaging over: (Top) the whole tissue section; and (Mid/Bottom) ROIs indicated in **Figure 2**. Peaks corresponding to OzID ions indicative of PC 34:1*n-*7 and PC 34:1*n-*9 are displayed as a fraction of total signature ions in the pie charts. Data were obtained using a modified Waters SYNAPT G2Si (operating at a mass resolving power of ˜15,000) . The precursor ion (*m/z* 782) was isolated using a 1 Th. isolation window, and OzID took place during transit of the mobility cell (15 ms). See **Figure S9** for an OzID fragmentation scheme and look up table for spectral interpretation.

To disentangle isobaric overlap and explore the distribution of lipid isomers, OzID was employed. Characterization by OzID not only reveals the location of double bonds within a lipid but can also distinguish monounsaturated and polyunsaturated lipids. MALDI-MS-OzID analysis of the prostate tissue section revealed the presence of only monounsaturated lipids, which eliminated 26 of the 30 possible species identified with the LIPID MAPS database. Of the remaining 4 species, 3 were unsaturated ether lipids (*i.e.*, plasmanyl/plasmenyl lipids) that could be excluded based on the absence of characteristic OzID fragments. Based on this analysis, PC 34:1 was assigned as the sole *monounsaturated* lipid sum composition within the selected mass window. Representative mass spectra, shown in **Figure 1**, highlight OzID product ions at *m/z* 700 and 672, revealing the presence of the lipid isomers PC 34:1*n*-7 and PC 34:1*n*-9, respectively. Using a co-ordinate sampling system, the abundance of these signature ions was then mapped across the tissue to visualize the spatial distribution of PC 34:1 isomers. Averaging the mass spectra over the whole tissue reveals an *n*-9 to *n*-7 ratio of ~90:10 (**Figure 1** top). Importantly, investigation into two individual regions of interest (ROI; **Figure 1** mid/bottom) reveals that *n*-7 and *n*-9 signature ions were found to vary independently. Because averaging the mass spectra across the whole tissue is analogous to data obtained from homogenised tissue lipid extractions, this example highlights the advantage of applying isomer-resolved techniques in MSI.

**Figure 2:**
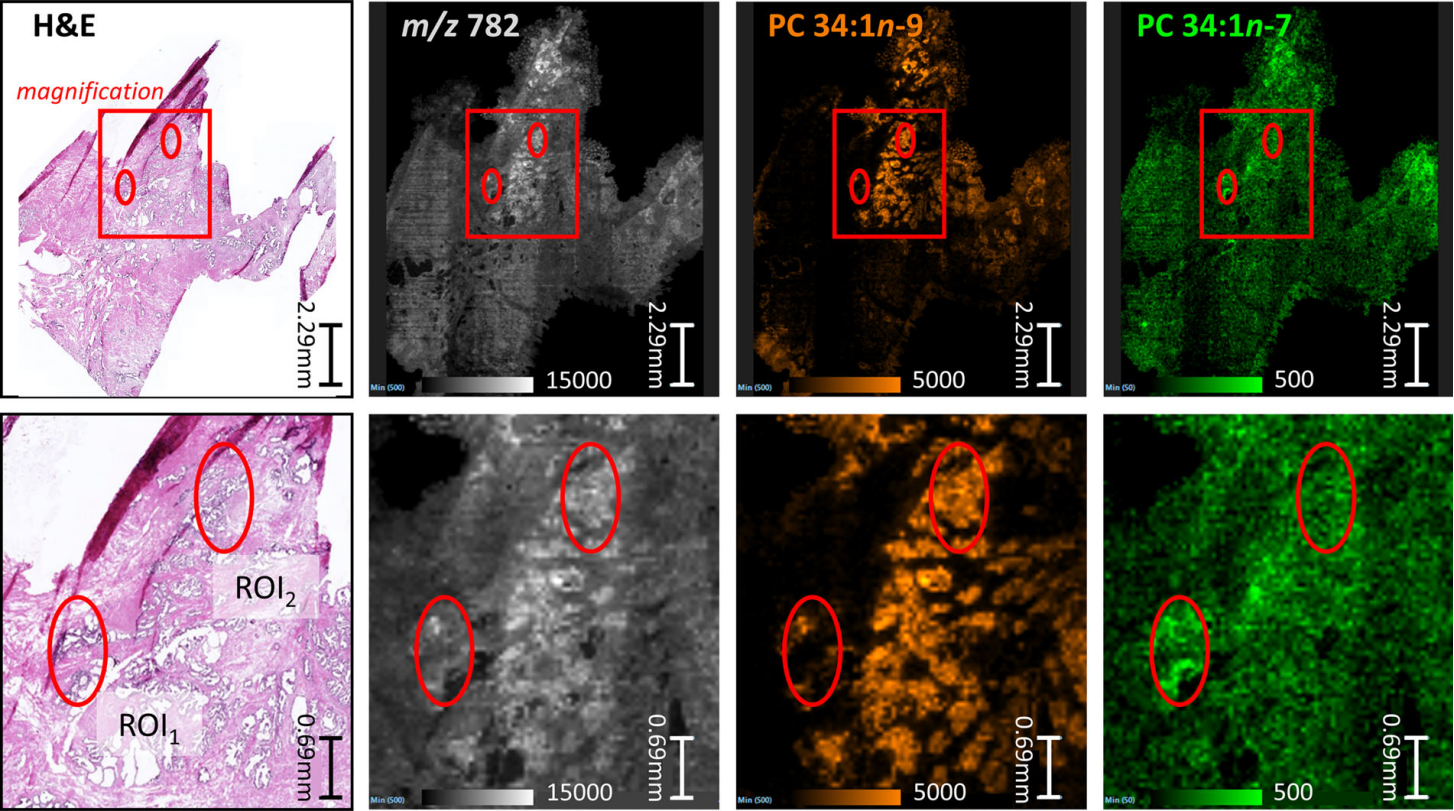
Comparison between serial sections of a prostate tissue following either routine staining (H&E) or MALDI-MSI-OzID. Top row: red box indicates the magnified region show in the bottom row, and ellipses provide comparative reference points. (Pink/purple) Prostate tissue H&E stain and micrograph with ROIs independently determined by two pathologists as inflammation (ROI_1_) or abnormal prostate epithelia (ROI_2_); (White) MALDI-MSI image showing the distribution of all product ions of *m/z* 782; (Orange) Isomer-resolved MALDI-MSI distribution of PC 34:1*n*-9 identified from OzID product ion at *m/z* 672; (Green) Isomer-resolved MALDI-MSI distribution of PC 34:1*n*-7 identified from OzID product ion at *m/z* 700. Each pixel within the MSI images is a spectrum similar to **Figure 1**, and thus each image show a product ion spectral intensity mapped across the tissues. Displayed product ion *m/z* was within 5 ppm of theoretical value.

To better visualize the isomer variation across the whole prostate tissue and any correlations between isomers and specific morphological features, MALDI-MSI-OzID images are shown in **Figure 2** and display: the tissue H&E, the distribution of the *m/z* 782 channel (white), and the distribution of PC 34:1*n*-9 (orange) and PC 34:1*n*-7 (green) based on the abundance of diagnostic OzID product ions. The two ROIs were selected from the H&E tissue stain based on noticeable difference in gland state and the presence of varying cell types (shown as red ellipses in **Figure 2**). ROI_1_ appeared to contain an atrophic gland surrounded by immune cells, and ROI_2_ was independently classified as benign intraductal prostate epithelia or as epithelia having abnormal features (malignant intraductal carcinoma of the prostate [IDC-P] or benign high-grade prostate intraepithelial neoplasia [HGPIN (*cf.* **Figure S1**). Formalin-fixed paraffin embedded (FFPE) tissue is the gold standard, and is routinely used for discriminating between these states, as the tissue architecture and cellular details are much better preserved compared to frozen sections. It is also challenging to distinguish these states in biopsy specimens, even with FFPE, given the small amount of tissue available for analysis. To assist reader comprehension throughout, areas of inflammation and immune cell (lymphocytes) infiltration will be referred to as ROI_1_ type tissues, while prostate epithelia and IDC-P/HGPIN will be referred to as ROI_2_ type tissues. Cross-referencing the H&E and isomer-resolved lipidomic images indicates that an increased abundance of PC 34:1*n*-9 is spatially associated with the ROI_2_ type, while an increased abundance of PC 34:1*n*-7 is associated with the ROI_1_ type. This difference in isomeric lipid distribution and subsequent disease-state association could be used to assist pathologist when examining patient needle-core biopsies for diagnosis.

### Calibration of MALDI-MSI-OzID for Lipid Isomers

**Figure 3:**
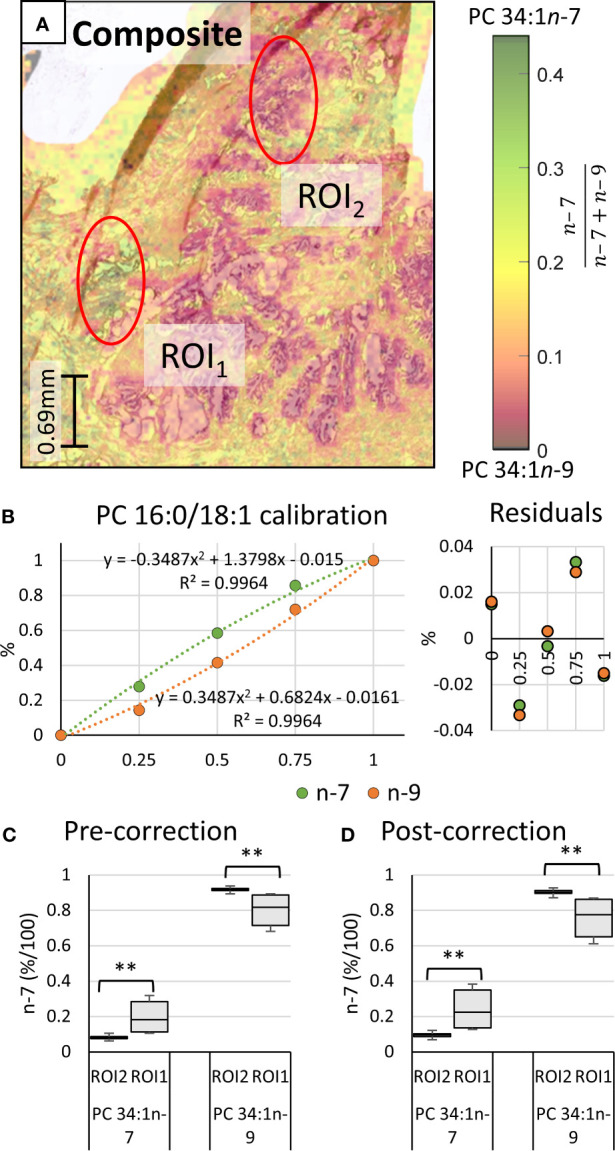
Haematoxylin and eosin stained tissue and calibration of mass spectral data to mol%. Comparison of two regions of interest (ROI) independently determined by two pathologists as inflammation (ROI_1_) or abnormal prostate epithelia (ROI_2_). **(A)** Overlay of the H&E stain and the PC 34:1*n*-9 and PC 34:1*n*-9 FDIs. Colour scale shows the fraction of n-7 (relative to total *n*-9 + *n*-7) from 0.0 (orange/black) to 0.45 (green/black). **(B)** Calibration curves and measurement residuals obtained from OzID analysis of reference standards PC 16:0/18:1*n-*7 and PC 16:0/18:1*n-*9. **(C)** The fractional ion abundance of isomer-specific OzID product ions corresponding to PC 34:1*n-*7 and PC 34:1*n-*9 displaying distribution of data across patient tissue ROIs (mean and standard deviation of 44 sampling regions). **(D)** The relative molar abundance of PC 34:1*n-*7 and PC 34:1*n*-9 in the ROIs, based on the calibration and correction with reference standards. Two-tailed paired Welsh’s *t*-test conducted, **p ≤ 0.01.

Underlying the ROIs indicated in **Figure 2** lies rich mass spectral data from which signature ion abundance differences between tissue features can be statistically explored. The mass spectra from each of the two ROIs highlight a significant variation in signal across the two regions (*cf.* **Figure 1** mid & bottom and **Figure 2** white), noting a higher maximum peak intensity in the ROI_2_ compared to ROI_1_. Because of variations in cell density within these regions (H&E), this observation indicates that changes in absolute lipid abundance are due to different tissue structures instead of variation in desorption and/or ionisation events. Looking past the absolute signal intensity and focusing on the relative abundance of isomer-specific OzID ions should instead provide an internally calibrated measure of changes in lipid metabolism. Using these ion abundances, the ratio of ion signals from PC 34:1*n*-7 to PC 34:1*n*-9 (relative to total PC 34:1) was mapped across the tissue in what is known as a fractional distribution image (FDI; where 0.0 and 1.0 represent hypothetical regions of pure *n*-9 and *n*-7 isomers, respectively) (29, 34). **Figure 3A** displays the overlay of the FDI and H&E images to draw visual correlations between isomer distributions and ROI type-1 and -2 tissues. Plotting these ratio-metric data within each ROI (**Figure 3C**) shows that the mean fractional abundance of PC 34:1*n*-7 signal decreases by ~0.116 (from 0.199 to 0.083) with a concomitant fractional increase in *n*-9 ion abundance within ROI_2_ type tissue. Statistical analysis revealed that the difference in isomer fractional abundance between these two ROIs was significant (p ≤ 0.01). Fractional differences were also observed across other, similarly classified ROIs (including patient replicates) and are shown in **Figure S2**. Although comparisons of the OzID product ion abundance reflects changes to the relative abundance of lipid isomers, it does not directly equate to relative molar contributions due to potential differences in detection efficiencies between the two isomers. For this reason, PC 34:1 reference standards were deployed to establish a calibration curve for *n-*7 and *n-*9 double bond isomers.

**Figure 4:**
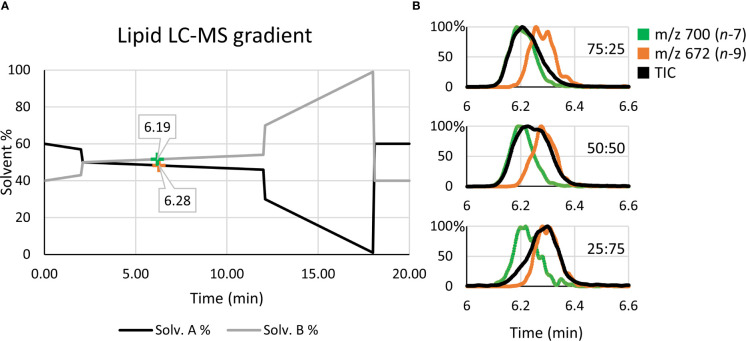
Liquid chromatography solvent gradient and elution profiles of isomeric reference standards. **(A)** Using OzID fragment XICs, the retention times for PC 16:0/18:1*n*-7 (green) and PC 16:0/18:1*n*-9 (orange) are mapped along the solvent gradient. **(B)** The use of OzID fragment XICs, such as m/z 700 (green) and m/z 672 (orange), resolve the quantitative accuracy issues arising from the poor isomer separation observed in the TIC (black).

Quantitative accuracy is reliant on the calibrant standards being structurally identical to the analyte (35). To generate the calibration (**Figure 3B**), high regiopurity PC standards (*i.e.*, the *sn*-2 unsaturated PC 16:0/18:1*n*-7 and PC 16:0/18:1*n*-9) were analyzed as these *sn*-2 regioisomers are more commonly found in mammalian cells (typically above 90%) (16). To ensure that small contributions of the *sn*-1 unsaturated isomers (*i.e.*, PC 18:1*n*-7/16:0 and PC 18:1*n*-9/16:0) did not significantly impact calibration of biological samples, the equivalent calibration curve was generated using analogous *sn*-1 unsaturated reference standards (see Supporting Information, **Figure S3**). To create the calibrations, liquid chromatography (LC) was implemented to allow for partial separation of the isomer reference standards before characterization and quantification *via* MS. **Figure 4** displays the solvent gradient used to achieve this challenging isomer separation (**Figure 4A**), and the peak resolution obtained from OzID extracted ion chromatograms (XIC; **Figure 4B** green/orange) and the total ion chromatogram (TIC; **Figure 4B** black). As is observed from the TIC, peak resolution between the isomers is poor, and thus TIC data would be unsuitable for quantitative calibration. Instead, unique OzID product ions eliminate the need for peak separation due to their isomer-specific presence. Creation of the calibration curves in **Figure 3B** was therefore achieved through comparisons of these characteristic OzID fragments of *sn*-2 unsaturated isomers.

To create both *sn*-1 and *sn*-2 calibration curves, relative OzID product-ion abundance (y-axis) was plotted against the fractional molar abundance of PC 34:1*n-*7 and PC 34:1*n-*9 (x-axis). Although it was hypothesized that the calibration would be non-linear due to competing collision- and ozone-induced dissociation processes (36), linear regression analysis was first explored. The linear fit revealed a high R^2^ value (0.9865), however investigation of the residuals found systematic trending, suggesting that OzID response calibration was better suited to a non-linear fit (see Supporting Information, **Figure S4**). Further, the limit of detection and limit of quantification for the linear calibration were y=0.14 and 0.41, respectively. Given that PC 34:1*n*-7 contributions range between 0.07 ≤ y ≤ 0.14 of the uncorrected isomer fraction, the lower limits of linear-calibration would be too large to distinguish specific tissue regions based on isomer ratios. For these reasons, a second order polynomial was instead fitted to the data (**Figure 3B**).

Regression analysis of the second order polynomial was shown to improve the fit (R^2^ = 0.9964) and abate trends in the residuals (*cf.* **Figures 3C** and **S4**). Individual residual values were also observed to reduce by 0.006-0.040 to give a standard error of the estimate (S_e_) of 0.022. Direct comparison of the instrument response fraction (*i.e.*, OzID product-ion abundance) to the known mole fraction revealed that differences between actual measurements and corrected values ranged from 0.015 (± 0.022) to 0.088 (± 0.022) with an average difference of 0.052 (± 0.022). Although the parabolic nature of the trendline might suggest that at low relative abundance of PC 34:1*n*-7 the instrument response will slightly underestimate its mole fraction, these differences fall within error values. Thus, the relative instrument response is approximately equivalent to the mole fraction. Nevertheless, calibration should be used where possible, to provide an explicit mole ratio for the isomers. Applying calculated correction factors to the tissue ROIs (**Figure 3D**) revealed that differences in the mole ratio between isomers were exacerbated compared to instrument response fractions. Comparative analysis showed that the mean mole ratio of PC 34:1*n*-7 increased by 0.147 (± 0.022) within ROI_1_, while ROI_2_ showed the concomitant increase of PC 34:1*n*-9 (0.147 ± 0.022). This presents that the apparent abundance of *n*-9 in ROI_2_ type tissues increased by an additional 0.030 when observing corrected mole fractions instead of raw instrument response. While instrument response was shown to be only slightly less sensitive to these differences in isomer abundance, correction of isomer signal to mole fractions subsequently translates data into biologically meaningful changes to lipid metabolism in tissues. Alongside these results confirming that isomer abundance differences between ROI type-1 and -2 tissues are indeed biological changes to lipid metabolism, the change to mol% also allows examination across and between without the need for an internal calibrant for normalization. Further investigation into other ROI 1 and 2 tissue types within the same patient tissue section revealed the relative mol% of PC 34:1*n*-7 decreased from 0.24 < x < 0.35 within ROI_1_ type features to 0.09 < x < 0.11 within ROI_2_ type features. Intriguingly, analogous analysis of tissue from a separate patient sample also revealed similar mole-ratio ranges of PC 34:1*n*-7 abundance to specific tissue types (ROI_2_ type: 0.32 < x < 0.35, ROI_1_ type: 0.10 < x < 0.12), which may indicate specific changes occurring to lipid metabolism in prostate cancers.

As mentioned previously, minor contributions of *sn*-1 unsaturated species may be present within the tissue samples. To ensure that these contributions were not significantly impacting mole ratio calibration, instrument response data from the *sn*-2 unsaturated reference standards were corrected using the calibration curve generated from the *sn*-1 standards. Although the trendline concavity can be observed to differ between *sn*-1 and *sn*-2 calibrations, this “cross-correction” of isomers revealed only a slight increase in S_e_, at 0.033. Given: (i) only a minor increase of S_e_ (0.022 to 0.033) is observed when correcting reference standards with the opposite calibration curve and (ii) the minimal presence of the *sn*-1 unsaturated isomers in mammalian cells (16), calibration of biological samples using an *sn*-2 unsaturate calibration curve will only minimally confound the correction to mole fraction. It should be noted that calibration curves were created using electrospray ionization (ESI), while tissue imaging experiments were conducted using MALDI. Nominally, both techniques are considered to be “soft” ionisation techniques, and thus observation of precursor ion fragmentation pathways appears identical.

### Lipid Isomer Ratio Based Classification of Prostate Tissues

**Figure 5:**
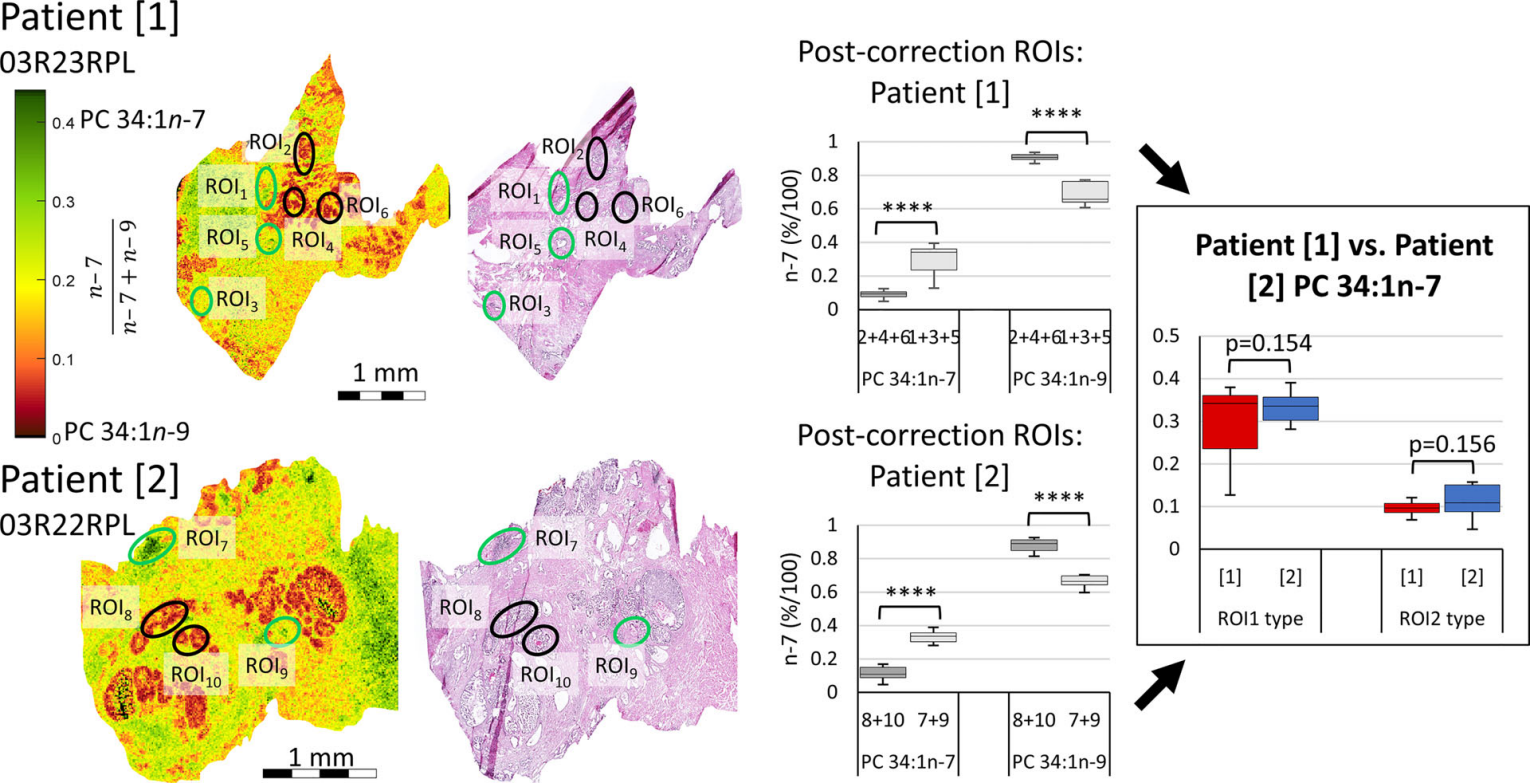
Comparison of isomer resolved lipidomics between two patient tissue samples. Isomer FDI (far-left) and H&E (mid-left) images showing correlation between tissue features and isomer distribution for two patient samples. FDI color scale bar ranges from 0.0-0.45 *n*-7 intensity to assist visual distinction and are based off biologically relevant abundances. ROI_1_ type tissues, containing a higher relative abundance of PC 34:1*n*-7, are indicated as green ellipses, while ROI_2_ type tissues, containing a higher relative abundance of PC 34:1*n*-9, are indicated in black. Product ion signal from the ROIs of either patient sample were corrected to mol%, grouped by ROI_1_ and ROI_2_ type similarity and compared intra-patient using a paired two-tailed Welsh’s *t*-test (mid-right). Patient samples were then compared inter-patient using a paired two-tailed *t*-test (far-right). (****p ≤ 0.0001).

While the experimental ordering of **Figure 3** used disease tissues to inform of ROIs warranting further investigation into isomer abundance, **Figure 5** instead investigates the isomer mol% of PC 34:1*n*-7 and *n*-9 across two patient tissues to observe whether mol% could be used independently stratify tissue state. Signature product ion signals from 10 ROIs were corrected to mol% using the calibration curve of **Figure 3B**. Post-correction ROIs were grouped by similarity (**Figure 5**, indicated with green and black ellipses) and were compared intra-patient (**Figure 5** mid-right). A paired two-tailed Welsh’s *t*-test was conducted and revealed significant difference between *n*-7 and *n*-9 mol% when comparing green and black ROIs (****p ≤ 0.0001). To compare if isomer mol% was patient specific or disease-state dependent, ROI data from either patient was grouped and compared, again using a paired two-tailed Welsh’s t-test (**Figure 5** far-right). This revealed that no significant difference could be established between the *n*-7 mol% (and hence *n*-9 mol%) within similar ROI types from different patient tissues. Based on the pathology assessment, this infers that (i) the increased mol% of PC 34:1*n-*7 appears to be a feature of human lymphocytes, and (ii) increased mol% of PC 34:1*n*-9 is a feature of epithelial lineage cells (HGPIN, IDC-P). Increased image resolution for **Figure 5** H&E and FDIs can be found in **Figure S5**. Three additional patient samples were obtained using the same analyses and can be found within the **Supplementary Information** (*cf.* **Figures S6**–**S8**). Statistical analysis using 28 ROIs across all 5 patients revealed clear distinction between ROI_1_ (lymphocytes) and ROI_2_ (abnormal epithelia) type tissues. Furthermore, two-way ANOVA analysis revealed that although patients displayed some differences between the *n*-7 and *n*-9 abundance ratios, no differences could be observed when comparing the *n*-7 and *n*-9 ratios of similar tissue types (0.41 F > 2.71 F crit; *cf*. **Figure S7**).

## Discussion

The data presented in **Figures 2**, **3** and **5** suggests that MSI can discern tissue components and states in prostate cancer specimens. While **Figure 2** indicates that isomer-resolved imaging can be used visually to give fast, qualitative results, **Figure 3** reveals that differences between tissue regions can also be assessed quantitatively and **Figure 5** displays that this appears robust from between patients in our preliminary analysis. Semi-quantitative investigation into the relative mole abundance of lipid isomers across prostate tissue can be used to visualize tissue inflammation and intraductal prostate epithelia that possibly show signs of pre-malignancy, while being independent from variation in overall lipid signal arising from variable desorption or ionisation efficiencies. Tissue studies of larger cohorts and the creation of critical isomer-abundance ranges are required and would then enable these numeric data to be paired with automated assessment protocols. When used in parallel with routine staining and tumor grading practices, these automated assessment protocols could provide clinicians with greater certainty about diagnoses and perhaps even inform on prognoses and progression. This would however require amendment to standard tissue handling workflows in pathology laboratories, as currently prostate tissue is not routinely processed to frozen sections.

**Figure 6:**
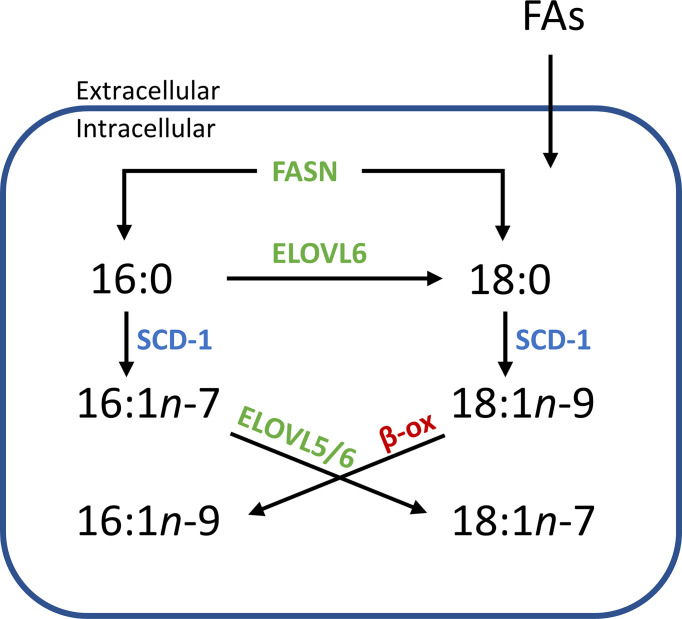
Simplified flow diagram of the origin and fate of fatty acid isomers depending on enzyme interactions. Increased abundance of one isomer over another in distinct tissue regions implies a systematic difference within an aspect of this scheme. *e.g.*, an increased relative abundance of *n*-7 isomers might imply that there is an increase of the starting substrate 16:0 (derived from intra/extracellular sources), while increases in *n*-9 isomers might indicate an increased abundance of 18:0 or an increased expression of ELOVL6. (SCD-1, steroyl- CoA desaturase; ELOVL, elongase enzyme; FASN, fatty acid synthase; β-ox, β-oxidation).

Alongside being used as a diagnostic tool, mapping lipid distribution across tissues can provide insight into underlying cellular metabolism. Within **Figures 2** and **5**, the distribution of PC 34:1*n*-7 and PC 34:1*n*-9 are observed to differ depending on tissue component and disease state. Considering a large majority of human PC 34:1 is made up by the PC 16:0_18:1 composition (16), this implies that the esterified monounsaturated FA is an 18:1 species and the distinctive distributions of each isomer within the tissue hint at confined metabolic changes. Given that the major product of *de novo* lipogenisis is 16:0 (palmitic acid), the formation of *n*-7 and *n*-9 double bonds is linked to modification of this saturated fatty acid. The schematic in **Figure 6** indicates two separate modification pathways that lead to the formation of 18:1 fatty acids from a 16:0 substrate. Although the desaturase SCD-1 (**Figure 6** blue) is viewed as responsible for the synthesis of both 16:1*n-*7 and 18:1*n*-9, the change in double bond location is dependent on the desaturase substate. It follows that differences in the ordering of elongation and desaturation reactions can lead to the formation of different 18:1 double bond isomers. For example, 18:1*n*-7 fatty acids are formed through direct SCD-1 (**Figure 6**, blue) desaturation of 16:0 substrates and subsequent elongation of 16:1*n*-7 by either ELOVL5 or ELOVL6 (green), while 18:1*n*-9 fatty acids are brought about from ELOVL6 (green) driven elongation of 16:0 to 18:0 (stearic acid) followed by SCD-1 desaturation (blue) substrates to yield 18:1*n*-9 (37). Therefore, the observed changes in the relative abundance of PC 34:1*n*-7 and PC 34:1*n*-9 within prostate tissue (**Figure 2**) indicate a discrete difference in the ordering of desaturation and elongation reactions, and suggests region-specific aberrations in the SCD-1 and ELOVL5 and/or ELOVL6 enzyme system(s). Indeed, recent work has shown that ELOVL5 is significantly upregulated within prostate cancer and depletion of the elongase lead to the generation of reactive oxidative lipid species and hindered cancer cell growth and proliferation (38). Previous research has implicated these enzyme systems in cancer, with overexpression of SCD-1 being linked to progression and growth of hepatocellular (39), renal (40), colorectal (41) and prostate (42) cancers. Likewise, a study on hepatocellular carcinomas indicated that patient tumors that were highly expressive of ELOVL6 were associated with shorter durations of overall survivability, while in a mouse-model inhibition of ELOVL6 increased survivability (43).

Concurrent with SCD-1 overexpression being related to cancer progression and growth, SCD-1 overexpression has also been shown to protect ovarian cancer cells from ferroptotic cell death (44). This study showed that cells transfected with SCD-1 siRNA were able to be rescued by the lipid peroxide scavenger, ferrostatin, revealing a relationship between the SCD-1 enzyme and ferroptosis. Interestingly, this research also revealed that ovarian cells primed to undergo ferroptosis (using the GPX4 inhibitor, RSL-3) were able to be rescued by the exogenous administration of the SCD-1 fatty acid product, FA 18:1*n*-9 (and to a lesser extent, FA 16:1*n*-7). A link between fatty acid isomer abundance and disease state has been suggested before, with researchers showing the malignant phenotype in prostate cancer cells contains a higher abundance of FA 18:1*n*-9 relative to that of the FA 18:1*n*-7 isomer (45). Combined, this suggests that SCD-1 overexpression is only partly responsible for the changes observed in cancer. Instead, its relationship with ELOVL expression and the subsequent abundance of *n*-7 and *n*-9 fatty acid products appears more impactful on cancer cell growth, progression and protection from cell death. This may be due to (i) the unique properties the lipid isomers impart on cellular membranes, (ii) the disruption created by the individual signalling capabilities of fatty acid isomers, or (iii) a combination of both. Indeed, an increased abundance of FA 18:1*n*-9 in cancer cells is interesting to consider in relation to the switch to FA catabolism as an energy source in cancer. As indicated in **Figure 6** (red), the *β*-oxidation (*β*-ox) of FA 18:1*n*-9 leads to the formation of FA 16:1*n*-9, a fatty acid that has been shown to signal potent anti-inflammatory effects (18, 19). Considering that cancer is largely orchestrated by pro-inflammatory cells and processes, the presence of this *de novo* synthesized, anti-inflammatory FA isomer would be intriguing in terms of attenuating a pro-inflammatory response. It is possible that a proportion of the PC 34:1*n-*9 distribution displayed in **Figures 2** and **3** can be ascribed to the PC 18:0_16:1*n-*9 isomer. Alas, determining the double bond location *and* fatty acid composition within intact lipid imaging experiments is outside the bounds of technology used in the present study, but this hypothesis provides a strong motivation for future technological development.

The complexity of pathological categorization of ROI2 type tissues in frozen sections highlights a potential role for molecular pathology. Notably, that these regions containing an increased mol% of PC 34:1*n*-9 (*cf.* **Figure 5**) were independently classified as benign prostate epithelia or as having abnormal features (HGPIN, IDC-P). As these prostate tissues were acquired from radical prostatectomy samples that contained histologically confirmed prostate cancer, the absence of adenocarcinoma in the specific tissue section being viewed indicates the tumor is located elsewhere in the organ. Given that previous research has revealed increases of *n*-9 isomers in cancer cells and tissues (45, 46), it is possible that strongly contrasting regions observed within the FDIs (*cf.* **Figure 5**) are evidence of pre-malignant epithelial cells arising from field effects from an out-of-view tumor. Further investigation would be required to determine the validity of this speculation, however. Nonetheless, this body of work reveals that determining the double bond isomer abundance of specific lipid species can be used to identify and distinguish between tissue components and states (*e.g.*, increased abundances of *n*-9 in (potentially pre-malignant) epithelial cells and increased *n*-7 in lymphocyte cells). As a first for isomer-resolved lipid imaging, double bond isomers were corrected to display mole fraction, which can be used to cross-examine different tissue samples without the need for an internal calibrant. Further, these mol% fractions can be informative about ongoing metabolic processes and potentially provide greater insight about prognostic outcomes of prostate (and other) cancers. Together, these results show that isomer-resolved imaging can potentially be a useful tool for clinical pathology, however larger cohort studies are required to determine the critical ranges of lipid isomer mole-ratios to establish association with pathological disease-states, including pre-malignancy from cancer field effects.

## Data Availability Statement

The datasets generated and/or analysed during the current study are available as a data archive from QUT Research Data Finder using the following DOI: 10.25912/RDF_1625715878910.

## Ethics Statement

The prostate tissues used throughout were obtained from radical prostatectomy with the ethical approval of the St. Vincent’s Hospital Human Ethics Committee and in accordance with Australian National Health and Medical Research Council Guidelines. The patients/participants provided their written informed consent to participate in this study.

## Author Contributions

RY, SE, MC and SB conceptualised the project and methodology. BC and AB collected MALDI-MSI data. EW prepared tissue samples and staining, and EW, BS and AP characterised tissues. RY compiled and analysed the data for visualisation and ran statistical analyses. BP installed OzID technology on all instruments. BP, SE, RH, MC and SB all provided funding and resources for the project. RY wrote the manuscript and SB revised and edited. All authors contributed to the article and approved the submitted version.

## Funding

This work was financially supported by the Australian Government through award of an RTP scholarship (to RSEY); the Australian Research Council through the Discovery Program (DP190101486) and the Linkage Program (LP180100238, partnered with Waters Corporation); the Prostate Cancer Foundation of Australia and the Australian Government Department of Health through a Movember Revolutionary Team Award; and, the Dutch Province of Limburg as part of the “LINK” program. SRE, APB. and RMAH acknowledge funding from Interreg V EMR and the Netherlands Ministry of Economic Affairs within the “EURLIPIDS” project (project number EMR23). BSRC and RMA acknowledge funding from the National Cancer Institute of the NIH, Grant No. ROI1 CA213492. SRE acknowledges funding from the Australian Research Council Future Fellowship Scheme (grant number FT190100082) and the Netherlands Organisation for Scientific Research VIDI scheme (grant number 198.011). MALDI-MSI OzID technology was developed through an industry linkage supported by Waters Corporation and the Australian Research Council (LP180100238). The funder had no direct involvement in this study.

## Acknowledgments

The project team acknowledge the Central Analytical Research Facility operated by the Queensland University of Technology for provision of instrumentation and training in support of this project; Avanti Polar lipids® for the provision of standards; and David L. Marshall for helpful discussions.

## Conflict of Interest

S.J.B. holds patents on ozone-induced dissociation technology (A method for the determination of the position of unsaturation in a compound, US8242439 and US7771943).

The remaining authors declare that the research was conducted in the absence of any commercial or financial relationships that could be construed as a potential conflict of interest.

## Publisher’s Note

All claims expressed in this article are solely those of the authors and do not necessarily represent those of their affiliated organizations, or those of the publisher, the editors and the reviewers. Any product that may be evaluated in this article, or claim that may be made by its manufacturer, is not guaranteed or endorsed by the publisher.
