# Supplementary material for: Isomer-Resolved Imaging of Prostate Cancer Tissues Reveals Specific Lipid Unsaturation Profiles Associated With Lymphocytes and Abnormal Prostate Epithelia
Source: Front Endocrinol (Lausanne). 2021 Aug 5;12:689600. doi: 10.3389/fendo.2021.689600 (PMC8374165; doi:10.3389/fendo.2021.689600)
Supplement: Supplementary file 1 [file DataSheet_1.pdf]

Supplementary information for:

Isomer- resolved imaging of prostate cancer tissues reveals specific lipid unsaturation profiles associated with lymphocytes and abnormal prostate epithelia

Reuben S.E. Young[1], Britt S.R. Claes[2], Andrew P. Bowman[2], Elizabeth D. Williams[3], Benjamin Shepherd[4], Aurel Perren[5], Berwyck L.J. Poad[1,6], Shane R. Ellis[2,7,8], Ron M.A. Heeren[2], Martin C. Sadowski[3,5] & Stephen J. Blanksby[1,6]*.

Affiliations

[1]School of Chemistry and Physics, Queensland University of Technology, Brisbane, QLD 4000, Australia.

[2]M4I, The Maastricht MultiModal Molecular Imaging Institute, Division of Imaging Mass Spectrometry, Maastricht University, Universiteitssingel 50, 6229 ER Maastricht, The Netherlands.

[3]Australian Prostate Cancer Research Centre - Queensland, Faculty of Health, Queensland University of Technology, Princess Alexandra Hospital, Translational Research Institute, Brisbane 4000, Australia.

[4]Department of Pathology, Princess Alexandra Hospital, Brisbane, QLD 4102, Australia

[5]Institute of Pathology, University of Bern, Murtenstrasse 31, CH-3008 Bern, Switzerland.

[6]Central Analytical Research Facility, Queensland University of Technology, 2 George St, Brisbane, QLD 4000, Australia.

[7]Molecular Horizons and School of Chemistry and Molecular Bioscience, University of Wollongong, Wollongong, New South Wales, 2522, Australia.

[8]Illawarra Health and Medical Research Institute (IHMRI), Wollongong, NSW, 2522, Australia

*Author to whom correspondence should be addressed: stephen.blanksby@qut.edu.au

Data availability statement

The datasets generated during and/or analysed during the current study are available as a data archive from QUT Research Data Finder using the following DOI: 10.25912/RDF_1625715878910

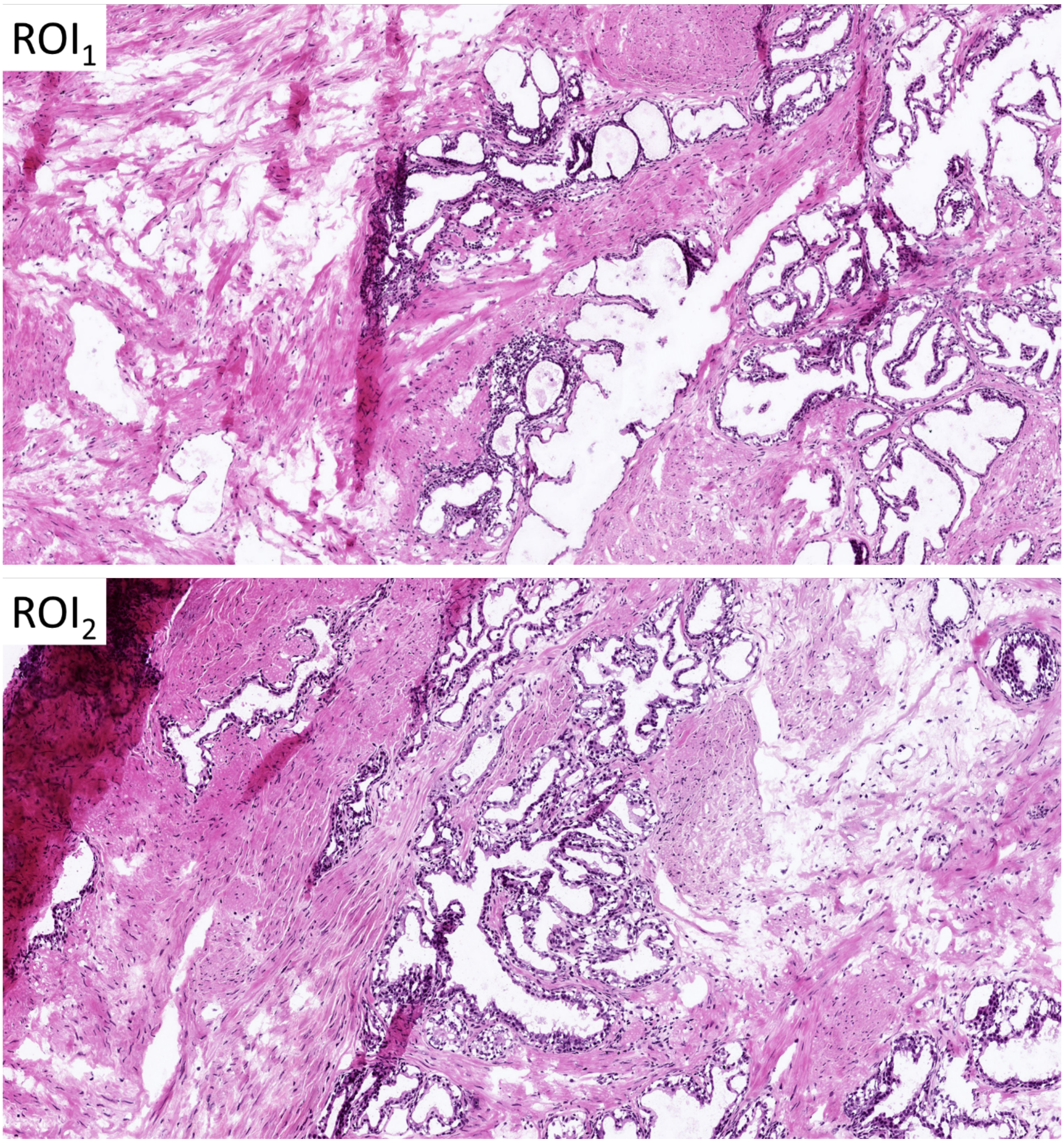

Fig. S1: High-resolution magnification of tissue ROIs relating to main text Fig. 2.
(Top) ROI_1 magnification of patient tissue 03R23RPL showing atrophic glands and immune cell infiltration to the region. (Bottom) ROI_2 magnification of patient tissue 03R23RPL showing prostate gland epithelia with possible signs of IDC-P or HGPIN.

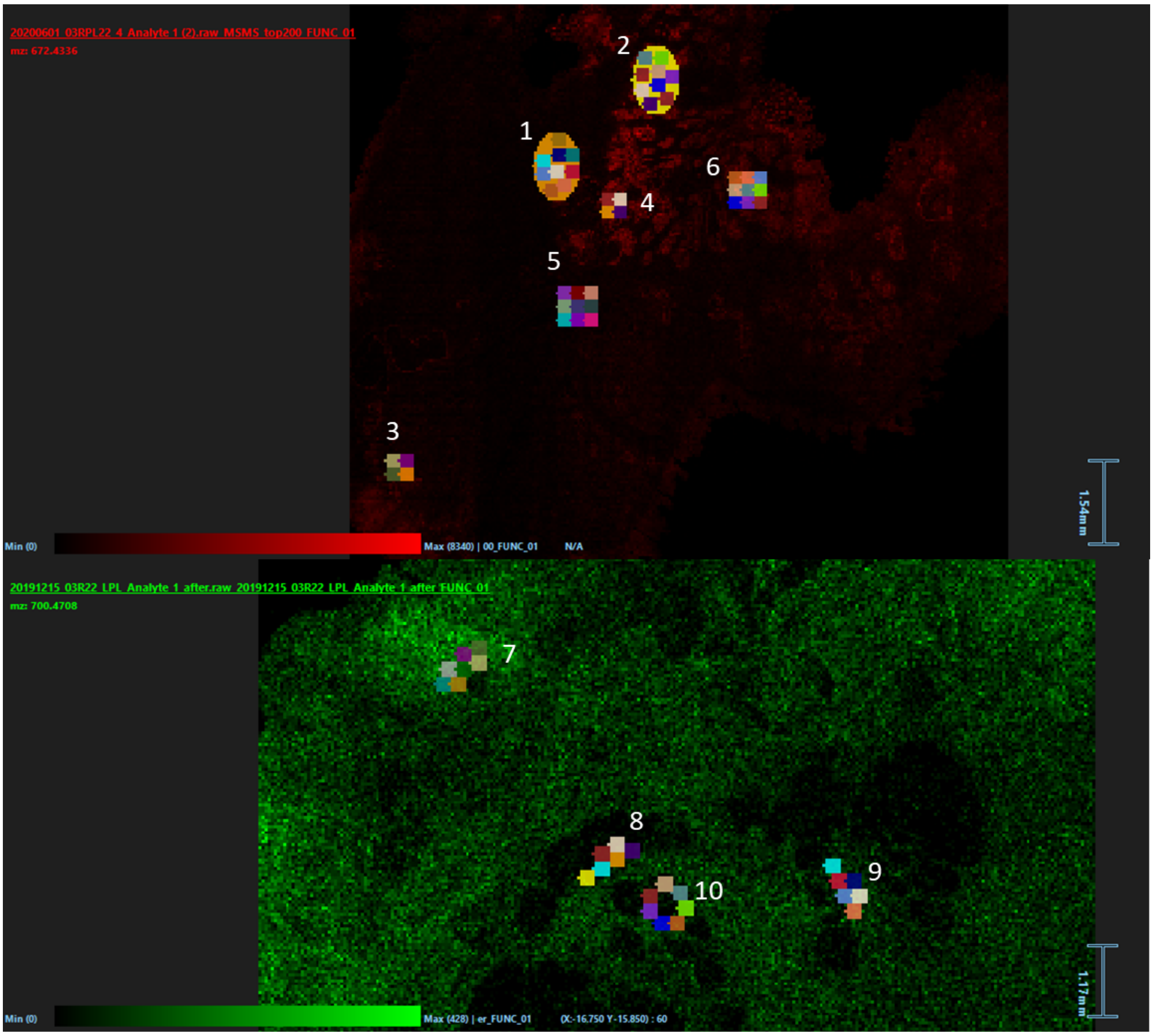

Fig. S2: Investigating multiple ROIs from the same patient tissue and secondary patient tissue. Top: The spectra underlying the elliptical ROIs 1 & 2 (indicated in Fig. 1-3) were sub-divided in into nine individual sub-ROIs averaging 6 x 6 pixels per each, allowing for the mean, standard error of the mean (SEM), and variance (var) to be calculated for the signals arising from *n*-7 and *n*-9 signature ions. A paired two-tailed Welsh's *t*-test was conducted to obtain statistical comparison between the regions (as seen in main text Fig. 3C) and revealed statistically significant difference between regions 1 & 2 (**p≤0.001269). For bio-replicate data for different tissues features from the same patient tissue, regions 3 & 4 (four sub-ROIs averaging across 6x6 pixels per each) and 5 & 6 (nine sub-ROIs averaging across 6x6 pixels per each) were compared and found to be statistical different (***p≤0.00094 and ****p≤2.2E-07, respectively). Bottom: The equivalent sub-sampling method and statistical analysis was also conducted using a separate patient tissue. Four regions across the tissue were chosen, with ROIs 1 & 2 (four sub-ROIs averaging across 6x6 pixels per each) being compared as well as 3 & 4 (nine ROIs averaging across 6x6 pixels per each). Again, statistically significant difference in the intensity of *n*-7 and *n*-9 signals was observed between the regions (****p≤1.45E-05 and ****p≤2.45E-05, respectively).

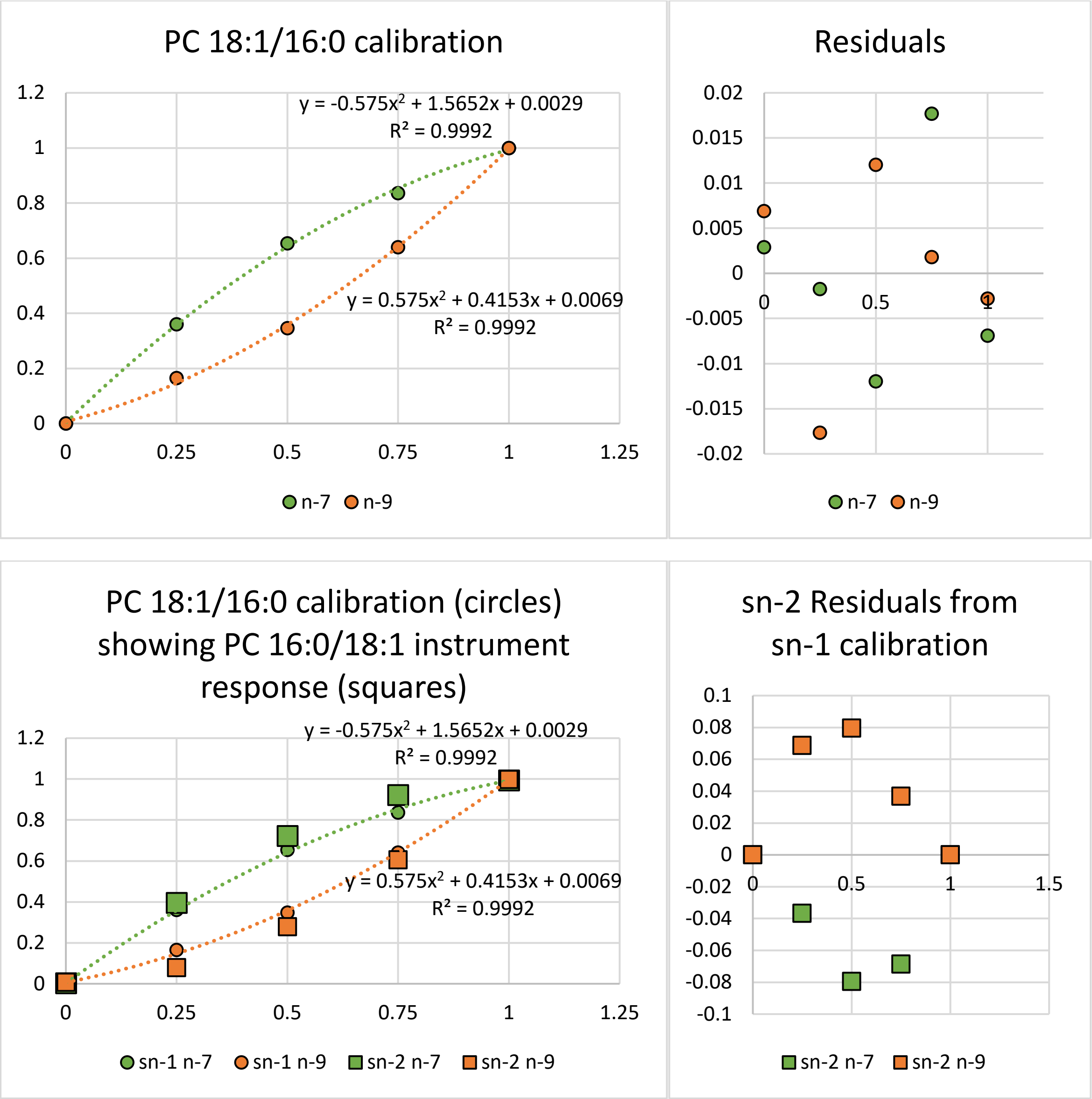

Fig. S3: *sn*-1 calibration and *sn*-2 measurements on *sn*-1 calibration

(Top -left) OzID response calibration curve generated using sn-1 monounsaturated (*i.e.,* PC 18:1/16:0) standards and a polynomial (x^2) regression analysis. R^2 value is decreased from the linear fit (0.9992) and (top-right) residuals now appear to be absent of systematic bias.

(Bottom-left) The OzID responses from the sn-2 monounsaturated standards (square-markers) plotted on the polynomial calibration curve generated from the sn-1 monounsaturated OzID responses. (Bottom-right) The *sn*-2 measurements do display systematic biasing in the residuals when correcting with an *sn*-1 calibration curve, which is to be expected due to fragmentation differences inherent to the MS method and lipid molecular structure. Because residual measurements fall within the error analysis, and biological contribution of sn-1 monounsaturated lipids to the isomer pool are low, this biasing can be discounted.

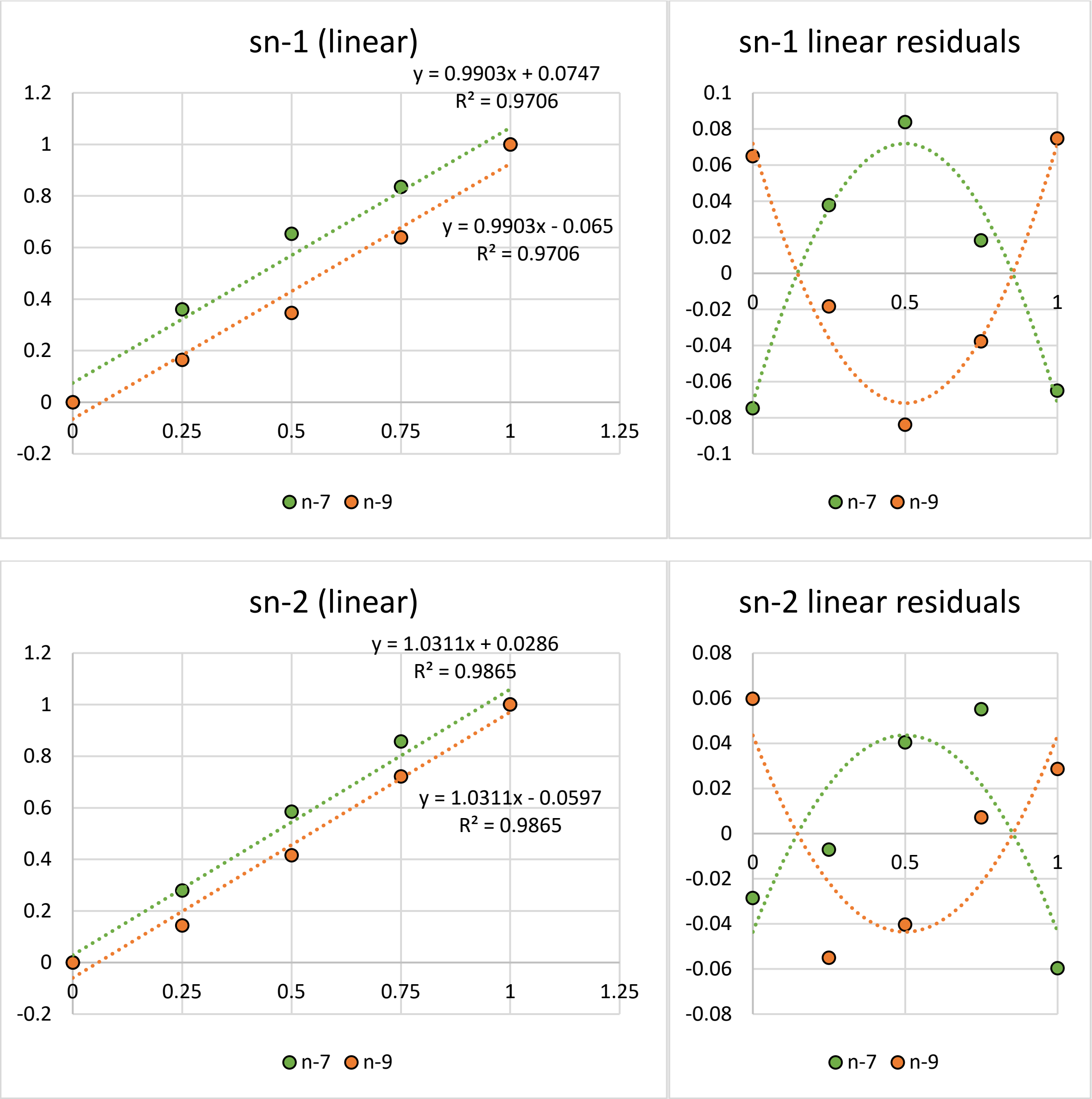

Fig. S4: linear calibration plots, residuals regression analysis for sn-1 isomer standards

(Top & bottom left) OzID response calibration curve generated using *sn*-1 monounsaturated (*i.e.,* PC 18:1/16:0) and sn-2 monounsaturated (*i.e.,* PC 16:0/18:1) standards and a linear regression analysis. The linear fit for both *sn*-1 and *sn*-2 monounsaturated standards displays a high R2 value (0.9865). The limit of detection and limit of quantification for the *sn*-2 chart can be seen to be y=0.14 and 0.41, respectively. (Right) Investigation of the residuals shows systematic trending for both *sn*-1 and *sn*-2 variants of PC 34:1*n*-7 and *n*-9 isomers, suggesting that OzID response calibration is better suited to a non-linear fit.

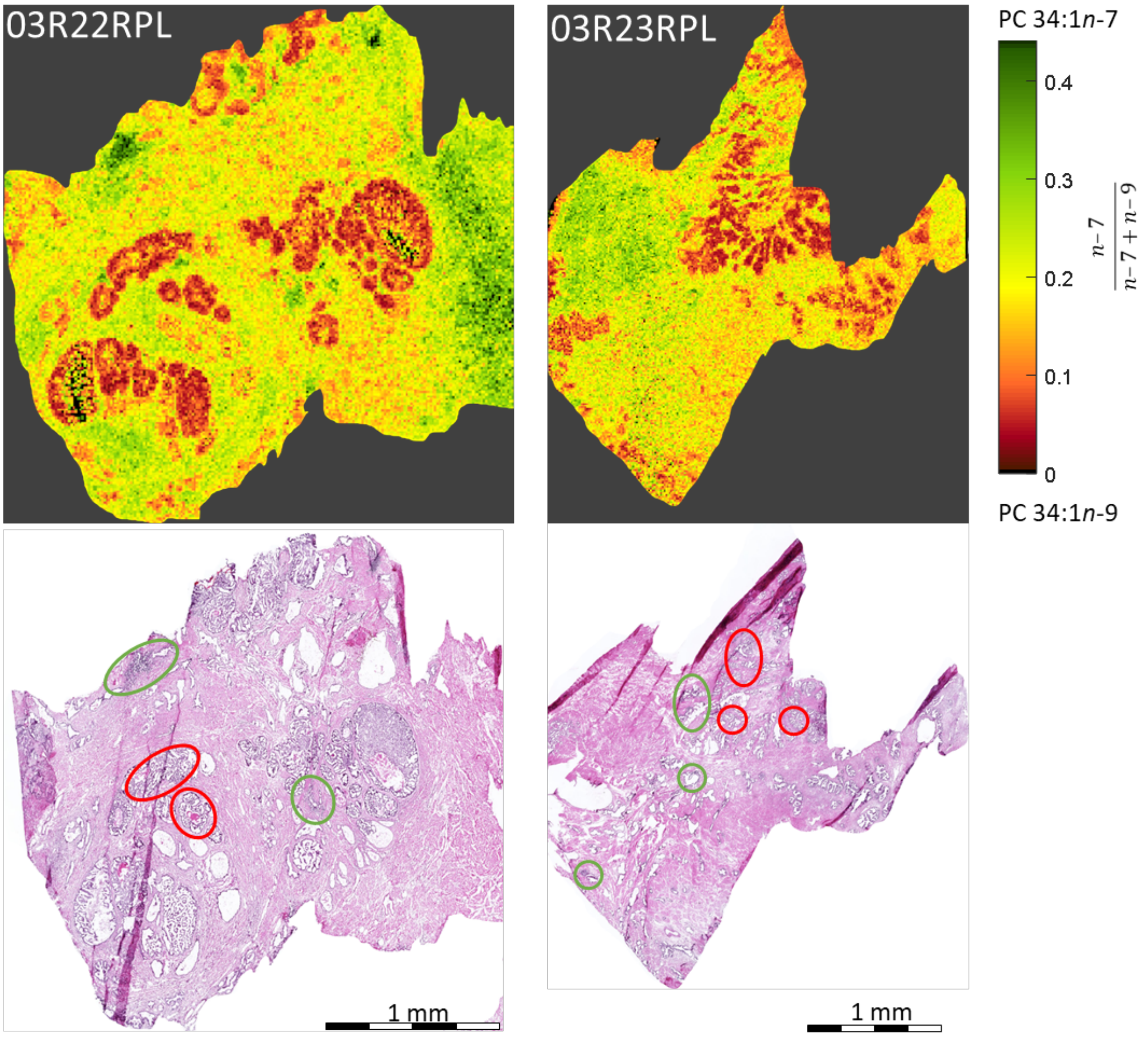

Fig. S5: Whole tissue images relating to main text Fig. 5.
(Top) Tissue FDIs for two patient samples showing the distribution of *n*-7 and *n*-9 isomer signals across the prostate tissues. Colour bar represents the biologically relevant range of *n*-7 normalised signal intensity (0.0 to 0.45), where 0.0 indicates the absence of n-7 and presence of n-9 (orange) and 0.45 indicates regions of comparatively high n-7 and lower n-9 (green). (Bottom) Tissue H&E stains for adjacent sections of prostate tissue with ROI type-2 and -1 tissue categorisation (indicated in red and green, respectively) being confirmed by two independent pathologists. Scale bars are relevant to both FDI and H&E images.

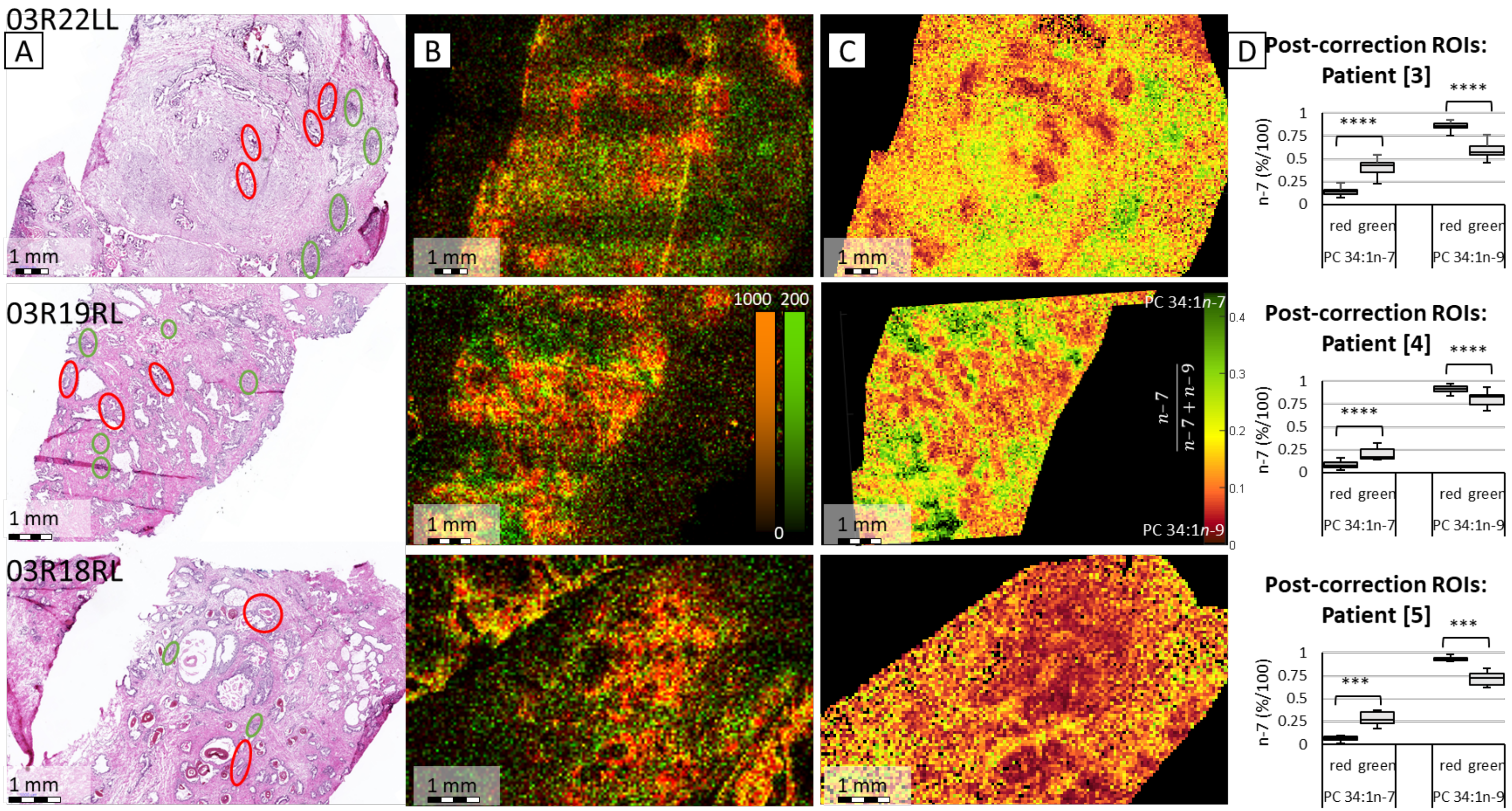

Fig. S6: Tissue replicate magnifications highlighting cell type regions and isomeric differences.
(A) H&A stained tissues highlighting regions of abnormal prostate epithelia (red ROIs) and immune cells (green ROIs). (B) MALDI-MSI-OzID product ion distribution showing the intensity of *n*-7 (green) and *n*-9 (orange) product ion mass channels across the tissues. (C) FDI image of *n*-7 and *n*-9 relative distribution across the tissues. (D) Box and whisker plots of all ROIs (including outside of magnification) for each tissue, with significant levels being determined by two-tailed students t-test (***p<0.001, ****p<0.0001).

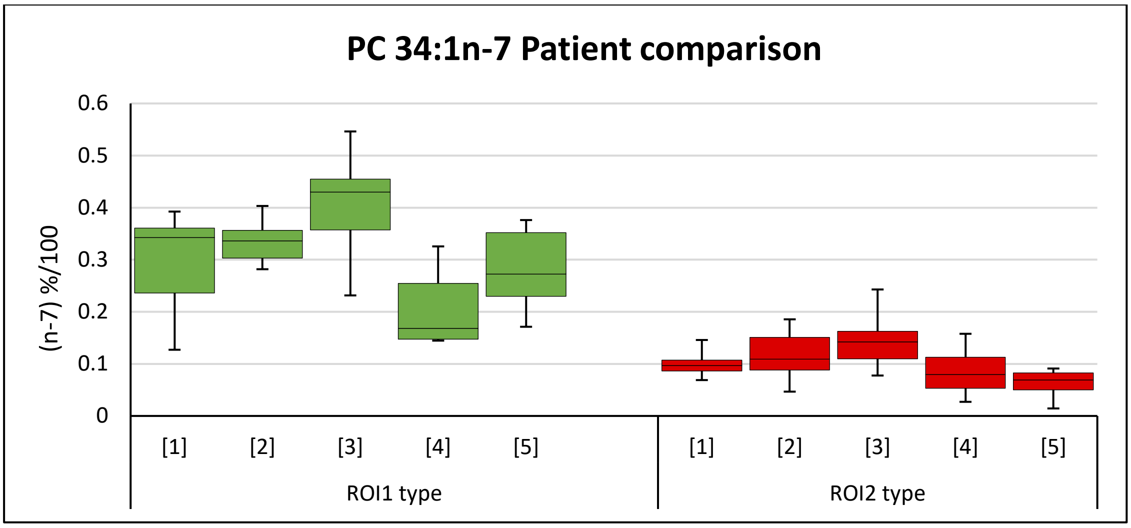

Fig. S7: Comparison of patient PC 34:1*n*-7 levels in tissue containing immune cells (ROI1 type) or abnormal epithelia (ROI2 type).

Box and whisker plots display the conventionally accepted values. Two-way ANOVA analysis reveals that there is a minor difference between the *n*-7 levels (3.39 F > 2.87 F crit) of patients, but also reveals that there is no discernible difference between the *n*-7 content of ROI types (0.41 F > 2.71 F crit).

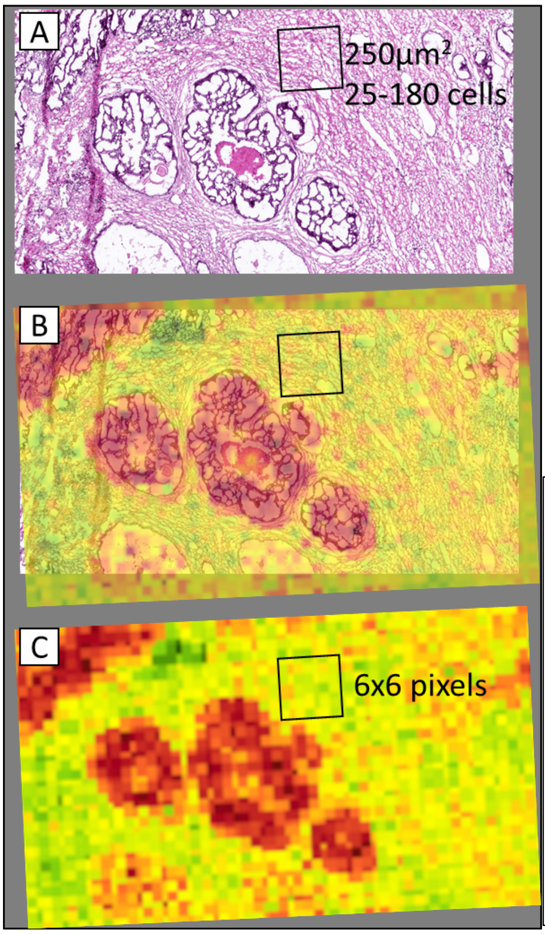

Fig. S8: MSI and H&E image alignment agreement, and analyte sampling size comparison.

(A) H&E stained tissue showing an example MSI sampling region, which depending on cell density equates to approximately 25-180 cells. (B) Composite image showing the MSI overlayed on the H&E. (C) MSI of tissue magnification showing pixel size and the number of pixels sampled for ROI creations. It should be noted that these two tissue sections are adjacent and are not images of the same tissue section.

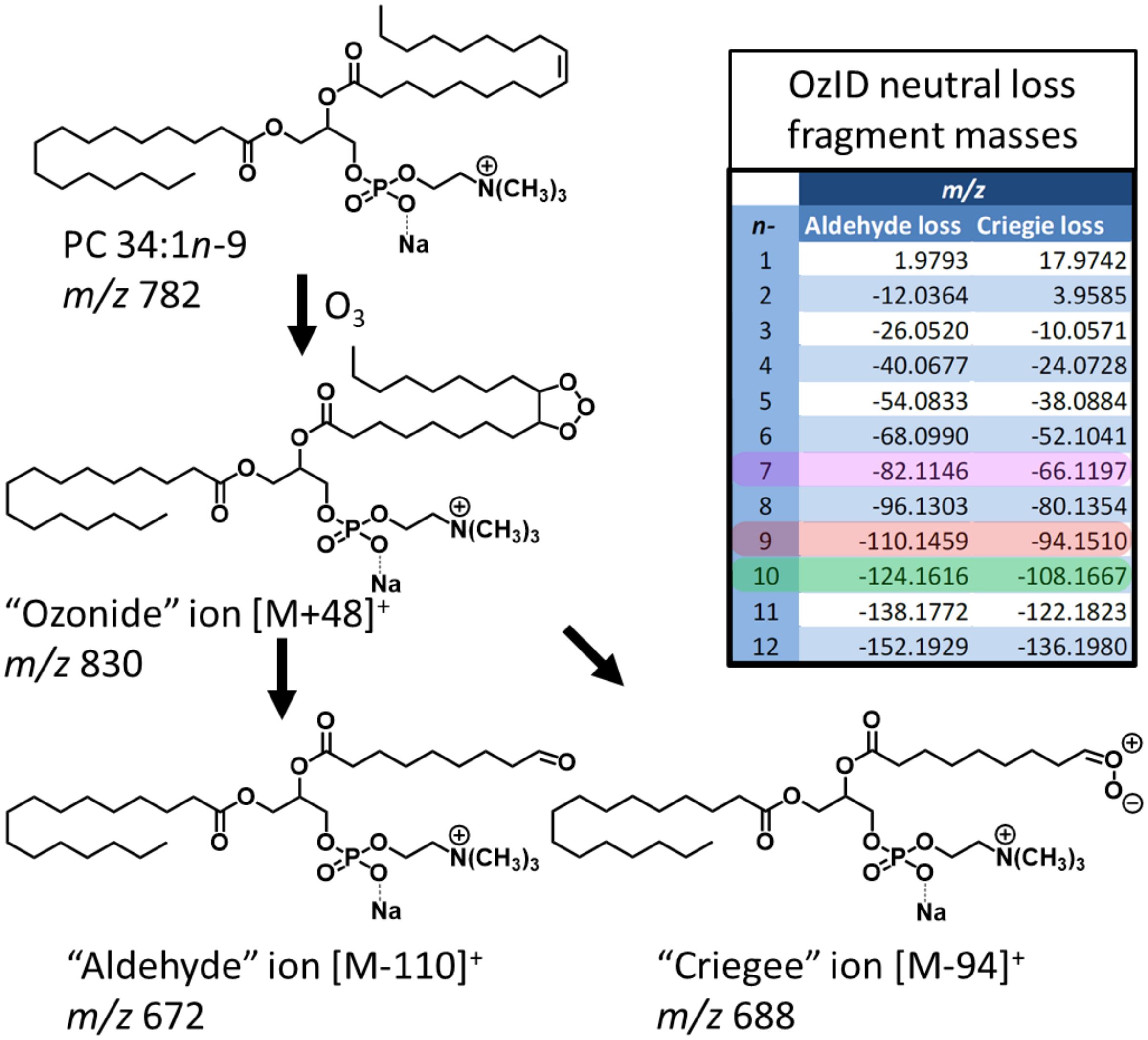

OzID neutral loss fragment masses

	m/z	
***n*-**	**Aldehyde loss**	**Criegie loss**
1	1.9793	17.9742
2	-12.0364	3.9585
3	-26.0520	-10.0571
4	-40.0677	-24.0728
5	-54.0833	-38.0884
6	-68.0990	-52.1041
7	-82.1146	-66.1197
8	-96.1303	-80.1354
9	-110.1459	-94.1510
10	-124.1616	-108.1667
11	-138.1772	-122.1823
12	-152.1929	-136.1980

Fig. S9: Ozone induce dissociation (OzID) fragmentation mechanism for the determination of intact lipid double bond positions.

(Left) The OzID fragmentation mechanism for PC 34:1*n*-9. After being trapped in the presence of ozone an ozonide intermediate is formed. Dissociation of this ozonide leads to the formation of two characteristic product ions. The *m/z* of the neutral loss is thus signature to the position of the double bond and theoretical value look-up table can be created for double bond location assignments. (Right) OzID double bond location look up table.
